# Mass propagation of *Juniperus procera* Hoechst. Ex Endl. From seedling and screening of bioactive compounds in shoot and callus extract

**DOI:** 10.1186/s12870-021-02946-2

**Published:** 2021-04-21

**Authors:** Abdalrhaman M. Salih, Fahad Al-Qurainy, Salim Khan, Mohamed Tarroum, Mohammad Nadeem, Hassan O. Shaikhaldein, Nadiyah M. Alabdallah, Saleh Alansi, Aref Alshameri

**Affiliations:** 1 Botany and Microbiology Department, College of Science King Saud University, P. O. BOX 2455, Riyadh, 11451 Saudi Arabia; 2 Department of Biology, College of Science, Imam Abdulrahman Bin Faisal University, P.O. Box 383, Dammam, Saudi Arabia

**Keywords:** Micropropagation, Podophyllotoxin, New compounds, *Juniperus procera*, Medicinal, Endanger plant, And DART-ToF-MS analysis

## Abstract

**Background:**

*Juniperus procera Hoechst*. ex Endl. is a medicinal tree in Saudi Arabia, primarily in the Enemas region, but it is locally threatened due to die-back disease and difficulties regarding seed reproduction (seed dormancy and underdeveloped embryonic anatomy, and germination rate < 40%). Hence, the alternative methods for reproduction of *Juniperus procera* are really needed for conservation and getting mass propagation for pharmaceutical uses.

**Results:**

In this manuscript, we articulated the successful in vitro shoot multiplication and callus induction of *J. procera* by using young seedling as explants and detected an important antibacterial and antitumor product. Explants were grown on different types of media with the supplement of different combinations of Plant Growth Regulators (PGRs) at different concentrations. The best media for shoot multiplication was Woody Plant Media (WPM) supplemented with PGRs (0.5 μM of IAA and 0.5 μM BAP or 0.5 μM IBA and 0.5 μM BAP). Whereas for callus induction and formation Woody Plant Media (WPM) with the addition of PGRs (0.5 μM 2,4-D and 0.5 μM BAP) was better than the Chu Basal Salt Mixture (N6), Gamborg’s B-5 Basal Medium (B5), and Murashige and Skoog media. The possibility of multiplication of *J. procera* in vitro creates significant advantages to overcome the difficulties of seeds dormancy for the reproduction of plants, conservation of trees, and getting mass propagation material for pharmaceutical studies. The shoot and callus extract of *J. procera* was detected using gas chromatography-mass spectrometry analysis and revealed more than 20 compounds related to secondary metabolites, which contained antibacterial and antitumor agents, such as ferruginol, Retinol, and Quinolone as well as confirmed by Direct Analysis in Real Time, Time of Flight Mass Spectrometry (DART-ToF-MS). Podophyllotoxin (PTOX) was detected in callus material by HPLC with sigma standard and confirmed by DART-ToF-MS and UV spectra.

**Conclusion:**

We successfully conducted in vitro shoot multiplication and callus induction from *J. procera* seedlings using WPM and a different combination of PGRs and, detected an important antibacterial and antitumor product such as ferruginol and podophyllotoxin. According to our findings, *J. procera* has become a new natural source of novel bioactive compounds.

## Introduction

The genus *Juniperus* L. is the second most prevalent group of conifer s on Earth [1]. *Juniperus procera* is coniferous evergreen tree or shrub of the Cupressaceae family. There are over 75 species of *Juniperus* [2]. *Juniperus procera* occurs in south Saudi Arabia in the Enemas region, and it is commonly called “Arar” in Arabic. *Juniperus procera* is indigenous to the mountains of eastern Africa from east Sudan to Zimbabwe, and southwest of the Arabian Peninsula, and it is widely spread throughout southern part of Saudi Arabia [3]. *Juniperus procera* is a source of natural drugs with potential anticancer, antimicrobial, insecticidal and antioxidant activities [4–6]. The antifungal activity of resin from *J. procera* was tested against *Pyrofomes demidoffii (*Lév*.)* Kotl*.* and an impressive result was observed [7]. It was reported that the leaves of *J. procera* is source of new flavonoid [3]. Besides that, fruits have medicinal values for headaches and curing skin diseases. Its resin was used in combination with honey as a stimulant and medicine to treat ulcers and liver diseases [8–10]. Numerous constituents of *J. procera* extract were detected using GC/MS analysis and reduced synthesis of aflatoxin B2 and G2 [11]. Moreover, it has various economic, social, and ecological values [12]. Juniper species have been used for many purposes including wood, landscaping and medicinal purposes [2, 13–15]. On the other hand, seed dormancy is a major hurdle for artificial regeneration of *J. procera* for economic and ecological importance [16]. In this context, it was reported that, some species of *Juniperus* have produced very small number of viable seeds or anatomically undeveloped [17]. Furthermore, the low number of fully developed seeds is one of the limitations of sexual reproduction in these trees [18, 19]. Mainly, because of drought, soil erosion, and increased runoff, the species has been declining progressively in many parts of the world [18, 20]. In some countries it is considered as an endangered tree [21]. The conservation strategies that have been used to date with traditional forestry techniques have not been satisfactory in many cases [16]. Since, very low potential for regeneration from seeds was reported. Thus, increasing attention has been paid to the possibilities offered by *in vitro* culture technology, which could be an alternative method for conservation and mass clonal propagation of different coniferous tree species [1]. In contrast, plant *in vitro* propagation is not limited by environment and seasons, and can overcome the problems of traditional breeding techniques. Hence, it become a reliable method for propagation of plants, especially, the production of endangered and rare species [22–26]. The first work on the *in vitro* propagation of juniper has been performed by Javeed and co-authors in 1980 [27]. A few studies have been done and reported on the subject of juniper *in vitro* propagation [2, 28–32]. Thus, *in vitro* propagation of juniper species should be exaggerated because, the micropropagation maybe the only alternative methods of the reproduction of this group of plants [1]. It should be prioritized for the possibility of conservation [33], and mass propagation for pharmaceutical uses. According to [34] *plant tissue* culture is an efficient approach to improve secondary metabolites production. Thus, due to the difficulty of *J. procera* regeneration through seeds, micropropagation technique can be employed to produce mass propagation material for pharmaceutical purposes, thereby natural regeneration through seeds in the wild will be maintained. Therefore, the primary objective of the study was the *in vitro* propagation of *J. procera,* to overcome the vegetative reproduction and seed regeneration problems. Secondly, the study aimed at the scanning of secondary metabolites, primarily, antibacterial and antitumor products for their economic importance.

## Materials and methods

### Plant materials and establishment of in vitro culture

In autumn 2019, young seedlings of four-year-old *J. procera* were collected from the botanic garden at the College of Food and Agricultural Sciences, King Saud University (origin from Elbaha region, southern part of Saudia Arabia). Firstly, the explants were washed with tap water for 5 min. Then, they were immersed in Clorox 25% (v/v) for 20 min and washed three times with sterile distilled water under the sterile flow of a laminar flow cabinet. Cutting about 1 cm contained at least one axillary bud from the terminal shoots of the seedlings of four-year-old of *Juniperus. procera* were used as explants for in vitro propagation.

### Media and plant growth regulators for shoot multiplication

**Fig. 1:**
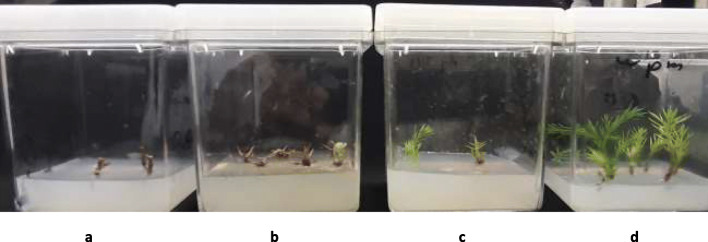
In vitro growth of *Juniperus procera* explants on different types of media (**a**): B5 (0.1 cm), (**b**): N6 (0.2 cm), (**c**) MS (0.4) and (**d**) Woody Plant Medium) (scale bar = 0.7 cm) after five weeks of growth

Four types of media were tested to evaluate the effects of Plant Growth Regulators (PGRs) and media compositions on shoot multiplication: (1) Woody Plant Medium (WPM) [35], (2) Murashige and Skoog (MS) medium [36], (3) Gamborg’s B-5 Basal Medium (B5 medium) [37], and (4) Chu Basal Salt Mixture (N6 medium) [38] (Fig. 1). Each was supplemented with combinations of PGRs, sucrose as carbon source (30 g/L), 7 g/L of agar, and the pH was adjusted to 5.7 before autoclaving at 121 °C for 20 min. Then, for each treatment four explants (three jars per treatment, in total 576 explants) were cultured under laminar conditions. Multiplication rate of the average number of shoots, the average number of branches, average of plant height and survival rate were recorded after five months of growth (Table 2).

### Callus induction

The explants (in total, 360) were sterilized as mentioned above. Then, inoculated into B5, MS, WPM, and N6 with different combinations and concentrations of PGRs (Table 2). In each treatment three replicates were used with three explant per jar. The jars were incubated in growth chamber at 25 °C ± 1, in the dark conditions, for three months.

### Preparation of shoot and callus extracts for gas chromatography-mass spectrometry analysis

100 mg shoot and callus of *J. procera* were lyophilized before placed in a pestle mortar and ground in 20 ml of 25 mM potassium phosphate buffer, with a pH of 7.0. The homogenate was transferred in a 100 ml conical flask and was shaken for 30 min at room temperature. Then, 20 ml of ethyl acetate was added, the mixture was incubated at room temperature for 5 min. Then, organic and aqueous phases were separated by centrifugation at 5000 rpm for 5 min. The organic phase was collected and evaporated in a vacuum. The residue was reconstituted with 1 ml of methanol and analyzed using gas chromatography-mass spectrometry (GC-MS 7890A; Agilent Technologies, USA) equipped with a 5975 mass-selective detector and a 7693 automated liquid sampler, fitted with a DB-5MS GC column (30 m length, 0.25 mm inner diameter, and 0.25 μm film thickness).

### Direct analysis in real time, time of flight mass spectrometry (DART-ToF-MS)

The extract samples were submitted to characterization by DART-ToF-MS analysis. The mass spectrometer instrument was AccuTOF LC-plus from JEOL (Japan). The volatile components of the extract were evaporated in a stream of helium heated at 250 °C, then ionized by the excited metastable helium atoms, before entering the ion source of the time of flight mass spectrometer. In the positive ionization mode, the molecules were mainly protonated without any fragmentation. The experimental conditions used for analysis of extract by DART-ToF-MS are listed in (Table 1).

**Table 1:** The conditions used for analysis of extract by DART-ToF-MS

Parameter	Value
Vacuum (Pirani gauge)	1.8 × 10^+ 2^ Pa
Vacuum (Analyzer)	1.3 × 10^−5^ Pa
Heater temperature	250 °C
Ionization mode	Positive
Injection gas	Helium
Ring lens voltage	4 V
Orifice 1 voltage	10 V
Orifice 2 voltage	5 V

### Chromatographic analysis of Podophyllotoxin

The identification of podophyllotoxin in callus and shoots of *Juniperus procera* was carried out on Agilent liquid chromatographic system controlled by G 4226A software. The column used for separation was a SB-C18 (1.8 μm, 4.6 × 150 mm). The mobile phase was consisted of MilliQ water (solvent A) and methanol (solvent B). The composition of the mobile phase was 30:70 (v/v). Detection was performed at 290 nm. Podophyllotoxin were initially identified by comparison of their retention times of standard and confirmed by UV spectra using SHIMADZU SPECTROPHOTOMETER (UV − 1800) in the range of 200-400 nm.

### Statistical analysis

Data from shoot proliferation and callus production were analyzed using one way of analysis of variance, which is a comparison based on a completely randomized design. A *P*-value < 0.05 was considered as statistically significant. The Duncan test was used to determine which treatments were different at (*p* < 0.05) using SPSS Version 20–32 bit (IBM, USA).

## Results and discussion

### Plant materials and disinfection

No problems were faced in relation to contamination during experiment. Many researchers have reported that there have been no problems with contamination during *Juniperus* spp*.,* In vitro propagation when different fragments of plants have been used as the initial explants [33, 39]. According, to literature review, the best method of juniper explants for establishing a culture contamination-free is plant material derived from in vitro growing explants, because they do not require any procedure of sterilization [40, 41] which are in agreement with our findings.

### Shoot multiplication

**Fig. 2:**
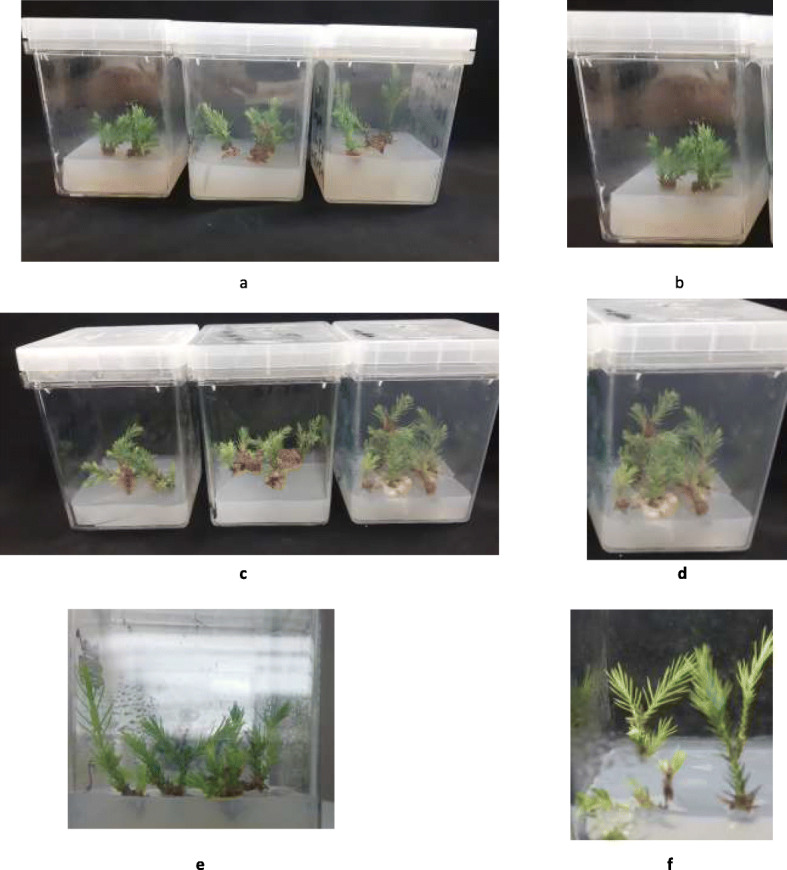
**a & b**: Effect of the combination of IBA and BAP on shoot multiplication of *Juniperus procera* on Woody Plant medium (WPM) (scale bar = 1.1 cm). **c & d**: Effect of the combination of IAA and BAP on shoot multiplication of *J. procera* on WPM (scale bar =1.0 cm). **e & f**: Effect of 2,4-D and BAP on shoot perforation of *J. procera* on WPM (scale bar = 0.75 cm) after five months

The effect of the culture of shoot multiplication was evaluated by comparing the response of shoots in different treatments: the morphological characters of shoot, survival rate, shoot number, shoot length, and the number of branches per shoot was evaluated for each treatment. The data on shoots multiplication were collected after five months of multiplication and by comparing explants response on all media. After three weeks of culturing, the color of explants in MS, B5, and N6 media changed from green to yellow (Fig. 1 a, b and c). Two weeks later, these cultures were excluded due to necrosis of explants, and the data were discarded from statistical analysis (Table 2). Shoots form *Juniperus phoenicea* L. growing on MS medium showed browned and necrotic zones reported by [2]. Data in Table 2 show the average number of shoots, average of shoot length growth, average of branches per shoot and survival rate on WPM with supplements of different combinations and concentration of PGRs. The explants successfully produced several new shoots over six weeks, which indicates that the best media was WPM. The highest average number of shoots regenerated from original explants was obtained on WPM with IAA (0.5 μM) and (0.5 μM) BAP (Table 2 and Fig. 2 c & d). While, the longest average shoot length was achieved on WPM with IBA (0.5 μM) and BAP (0.5 μM) (Table 2 and Fig. 2 a & b). Whereas, among 2,4-D and ABP combinations on WPM, (1.0:1.0) concertation was the best (Table 2 and Fig. 2 e & f). Shoots induction from juniper species explants ranged between 4 to 12.9 per explant for *Juniperus navicularis* Gand. and *Juniperus thurifera* L. respectively, reported by [31, 39]. In our study, the best shoots induction per explant about 14 by using WPM with IAA (0.5 μM) and BAP (0.5 μM) (Table 2 and Fig. 2 c & d). Khater [31] who has been reported that, the best elongation of shoots of *Juniperus thurifera* L., has been achieved on WPM with supplemented of 0.5 mg L^− 1^ of BAP and 0.25 or 1 mg L^− 1^ of 2,4-D. We observed that the combination of IBA and BAP or IAA and BAP had an important effect on vigorous growth, the development of shoots, survival rate, and elongated shoots compared with other PGRs combinations on WPM. Thus, is a significant point for mass propagation and conservation of this species. No report was found in the literature regarding *J. procera* shoot multiplication in vitro. In general, juniper species respond to low levels of cytokine and auxin [30]. For example, the concentration of BAP at 0.5 mg L^− 1^ in WPM was the best for the shoots multiplication of three juniper species (*Juniperus excels M.Bieb.*, *Juniperus horizontalis Moench.* and *Juniperus chinensis Roxb.)* reported by [2].

**Table 2:** Effect of plant growth regulators combinations and Woody Plant Media on shoot proliferation of *Juniperus procera* after five months of growth

Concentrations of PGRs (μM)	Average of shoots number	Average number of branches (shoot^**− 1)**^	Average of shoots length (cm)	Survival rate (%)
2,4.D + BAP (00: 0.00)	3.00 ± 0.00^ab^	1.67 ± 0.33^c^	2.83 ± 0.16^ab^	72.0
2,4.D + BAP(0.25: 0.25)	2.67 ± 0.33^b^	1.33 ± 0.33^c^	2.33 ± 0.33^b^	66.0
2,4.D + BAP (0.25: 0.5)	2.00 ± 0.57^b^	1.67 ± 0.33^c^	3.00 ± 0.57^ab^	50.0
2,4.D + BAP (0.25: 1.0)	2.66 ± 0.33^b^	1.67 ± 0.33^c^	2.83 ± 0.16^ab^	66.0
2,4.D + BAP(0.5: 0.25)	2.33 ± 0.66^b^	2.33 ± 0.33^c^	2.33 ± 0.33^b^	72.0
2,4.D + BAP (0.5: 0.5)	2.00 ± 0.57^b^	1.33 ± 0.33^c^	2.17 ± 0.16^b^	50.0
2,4.D + BAP (0.5: 1.0)	2.33 ± 0.33^b^	2.00 ± 0.00^c^	2.67 ± 0.16^b^	58.0
2,4. D + BAP (1.0: 0.25)	2.00 ± 0.57^b^	1.33 ± 0.33^c^	2.33 ± 0.33^b^	50.0
2,4.D + BAP (1.0: 0.5)	2.67 ± 0.33^b^	1.67 ± 0.33^c^	2.17 ± 0.16^b^	66.0
2,4. D + BAP (1.0: 1.0)	3.00 ± 0.00^ab^	1.67 ± 0.33^c^	2.33 ± 0.33^b^	72.0
IAA + BAP (0.5: 0.5)	4.00 ± 0.00^a^	14.00 ± 1.7^a^	4.00 ± 0.57^ab^	100
IBA + BAP(0.5: 0.5)	4.00 ± 0.00^a^	10.67 ± 2.6^b^	5.33 ± 0.88^a^	100

The data are presented the average of shoots, branches per shoot, average of shoot length ± standard error, and Survival rate (%).

^a,b,c^ Means within the same column with different superscripts differ significantly (*P* < 0.05)

### Callus induction

**Fig. 3:**
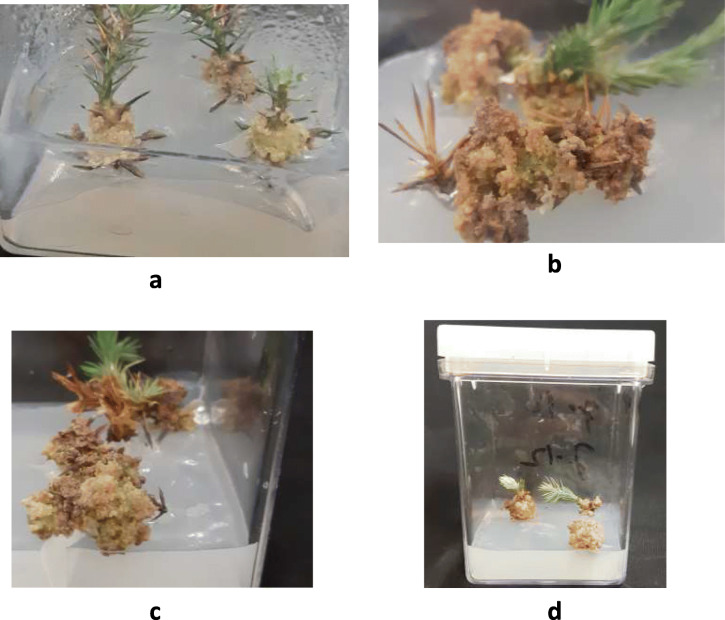
**a, b & c**: Effect of the combination of (2,4-D and BAP – 0.5: 0.5) on callus induction and formation from *Juniperus procera* on Woody Plant medium (WPM) (scale bar = 0.1, initiation of callus, while b& c = 0.5 cm), **d**: Effect of the combination of (2,4-D and BAP; 1.0:1.0) on callus induction and formation from *Juniperus procera* on MS medium (scale bar = 0.2)

Data in Table 3 show the influence of PGRs supplemented in WPM, B5, N6 and MS medium. The earliest sign of callus induction was noticeable after five weeks of incubation (Fig. 3 a). The amount of produced callus, percentage of calli induction was significantly different among treatments, for example, the produced amount of calli per treatment was ranged between 0.4 to 4.6 g. Which indicate that, among each treatment, the best callus type and callus induction rate (4.6 g) was in WPM with the addition of 0.5 μM BAP and 0.5 μM 2,4-D (Table 3 and Fig. 3 b, c) and less effective callus induction rate (0.4 g) was obtained using WPM supplemented with (0.25 μM) 2,4-D and (1.0 μM) BAP. While, in MS media, one combination (1.0 μM BAP + 1.0 μM 2,4-D) out of all PGRs combinations was induced callus 1.0 g (Table 2 and Fig. 3 d). Whereas, N6 and B5 media with the same PGRs combinations did not induce the callus and was excluded. The best callus quality from *J. thurifera* was achieved on WPM supplemented with either 0.5 mg L^− 1^ BAP + 0.25 mg L^− 1^ 2,4-D or 0.25 mg L-1132 BAP + 0.25 mg L^− 1^ 2,4-D [31]. Moreover, the best induced callus from *Juniperus* species *(Juniperus excelsa, Juniperus horizontalis, and Juniperus chinensis)* is obtained in WPM medium with 0.50 mg/L of BAP and 0.50 mg/L, 2, 4-D [28]. In turn, in coniferous species, an appropriate ratio of auxin and cytokinin is requested to stimulate the calli from explants reported by [1]. Although, Muranaka [42] stated that, callus was induced from *Juniperus chinensis* after application of higher concentrations of NAA (3.0 mg L^− 1^) and KIN (0.2 mg L^− 1^). The color of produced calli changed to a brownish but, sub-culturing over 20 days eliminated this problem. It is known that the induced calli may quickly turn brownish in juniper species [1].

**Table 3:** Effect of plant growth regulators combinations and different types of media on callus formation of *Juniperus procera*

BAP (μM) 2,4-D (μM)		Callus formation				Callus weight (g)
		MS	WPM	B5	N6	
0.0	0.0	–	–	–	–	0.0
0.25	0.25	–	–	–	–	0.0
0.25	0.5	–	*	–	–	1.0
0.25	1.0		*		–	0.4
0.5	0.25	–	–	–	–	1.0
0.5	0.5		***	–	–	4.6
0.5	1.0		**			3.1
1.0	0.25		–	–	–	0.0
1.0	0.5		*	–	–	1.0
1.0	1.0	*	–	–	–	1.0

---: no callus induction, *: = Poor, **: Fair, ***: Good.

### Gas chromatography-mass spectrometry analysis of shoot and calli of *Juniperus procera* extract

**Fig. 4:**
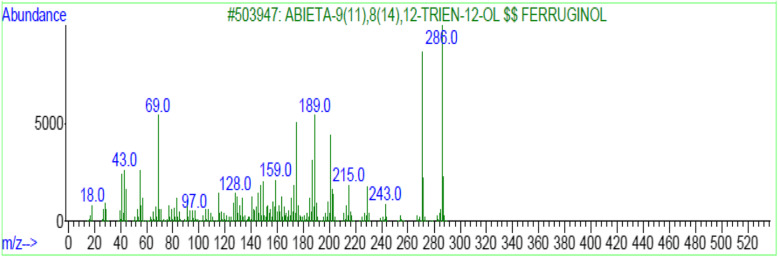
GC-MS of *in vitro* shoot and callus extract of *Juniperus procera* showing the ferruginol spectrum

In general, plants produce secondary metabolites as a protection mechanism against biotic and abiotic stress. On the other hand, it has been showed that, the in vitro propagation is a tool for secondary metabolites production, that can ultimately provide a continuous, reliable source of bioactive compounds. Moreover, a rapid production of important secondary metabolites can be achieved through micro-propagation technique [34, 43, 44]. The identification of the constituents in the extract was performed using commercial libraries and comparison of mass spectra, matches percentage, and retention times of reference compounds. GC-MS analysis of shoots and calli of *J. procera* extract revealed more than 20 constituents related to secondary metabolites (Table 4). But, variation has observed between shoot and calli extract (Table 4). These detected compounds are containing antimicrobial and antitumor agents, one of which is ferruginol (Fig. 4). The typical positive ion spectra obtained from calli and shoots of *J. procera* with molecular formula (C_20_H_30_O ± H) and m/z 286 ion was detected in calli and shoots (Fig. 4). Which is closely matches ferruginol (C_20_H_30_O, mol. wt 286.5) and corresponds well with NIST Standard Reference Database 1A [81]. In accordance, the peaks generated by ferruginol are normally 285 and 301 [82]. Ferruginol is a diterpene phenol, has received attention recently, for its pharmacological properties, including antibacterial, anti-tumor, gastro-protective and cardio-protective effects [66]. In addition, ferruginol inhibits the growth rate of cancer cells [67]. Moreover, ferruginol compounds tested against malaria strains 3D7 and K1 and some structure activity relationships were identified for antimalarial activity [68]. The second important compound is retinol (vitamin A). Retinol belongs to a family of endogenous natural retinoids and is a precursor for the synthesis of retinoic acid and retina. Retinol has playing an effective role in treating aging and photoaging, recounted by [83]. The third important compound is Quinolone, which is an antibiotic containing a bicyclic core structure [84]. Moreover, it has been reported that Quinolones are a class of antibactericidal and antibiotics with broad-spectrum activity, which can inhibit both Gram-positive and Gram-negative bacteria [75]. Although *J. procera* has been investigated widely as a source of natural drugs with anticancer, antioxidant, insecticidal potential and antimicrobial activities [6–8]. No available report has been found in literature related to *J. procera* in vitro propagation and secondary metabolites detection. The screening of natural *J. procera* extract reflect the presence of 46 secondary metabolite reported by [11]. It’s totally different from the constituents which revealed by the present study. While [83] has mentioned that the stem bark of *J. procera* grown in Saudi Arabia has yielded several antimicrobial diterpenes such as ferruginol. Moreover, two known abietanes (totarol and ferruginol) have been identified from berries of *Juniperus procera*, *Juniperus excelsa* and *Juniperus phoenicea* [45]. Besides the most important bioactive compounds described and highlighted above. The others phytochemical constituents detected in the shoots and callus extract of *Juniperus procera* and their activities are presented in Table 4.

**Table 4:** Some phytochemical constituents of in vitro shoots and calli of *Juniperus procera* detected by GC-MS and their biological activity

Shoot-Compounds	RT (min)	Activity	Callus - compounds	RT (min)	Activity
Cyclononasiloxane	27.214–31.660	Antibacterial [45]	2,4,6-Cycloheptatrien-1-one	18.406	Bacteriostatic and bactericidal [46]
S-Indacene-1,7-dione	33.548	Antifungal [47]	1H-Cycloprop [e]azulene	21.259	Analgesic and anti-inflammatory [48]
Perylene	34.252	Potentially mutagenic and carcinogenic [49, 50]	1H-Benzocycloheptene	21.259	Anti-tumor, anti-inflammatory, and antimicrobial [51]
Benzoic acid	35.813	Antimicrobial [52], ant hepatotoxic [53] and fungitoxic [54]	Cyclononasiloxane	27.214	Antibacterial [45]
1Phenanthrenecarboxaldehyde	36.677	Antimicrobial [55]	Kaur-16-ene	30.905	Anti-inflammatory [47]
Retinol	36.677	Photoaging and dermatologic disorders [56–58]	Podocarpa-6,8,11,13-tetraen-12-ol	30.905	Algicidal [59]
2-Phenanthrenol	38.891	Antimicrobial [60]	Phenanthrene	33.548	Anti-inflammatory, antiallergic, antimicrobial,cytotoxic, antiplatelet aggregation and phytotoxic [61–64]
n-Octanoic acid	39.118	Anti-inflammatory and anti-seizure [65]	S-Indacene-1,7-dione	33.548	Antibacterial [60]
Ferruginol	39.227–39.865	Antibacterial, antimalarial and antitumoral [62–64]	Adipic acid	34.009	a precursor for Nylon-6, and 6 polymer [61]
Prasterone	40.091	vulvovaginal atrophy treated [62]	Desomorphine	36.677	Analgesic [63]
n-Nonadecanoic acid	40.586	Antimicrobial [64]	Benz [c]acridine,	38.254	Antibacterial [69, 70]
9(1H)-Phenanthrenone	41.02	Antifungal [71]	Acetamide	38.254	Antifungal [71], Anti-inflammatory and analgesic [72]
2,6-Phenanthrenediol	42.507	Antimicrobial [73]	Podocarpa-8,11,13-triene	38.480	Anti-inflammatory [74]
Quinolone	43.329	Antibiotic [71, 72, 75]	Phenanthrenol	38.480	Drug precursor [76]
			Fluorophenol	38.623	antibacterial [77]
			Ferruginol	39.277–39.865	Antibacterial, antimalarial and antitumor [66–68]
			2-Phenanthrenol	39.277	antimicrobial [60]
			Acetamide	39.705	Anti-inflammatory and Analgesic [78]
			Benzene(1-nitro-4-(phenylmethyl)	39.865	inhibitor [78]
			Cyclopenta	40.527	Antitumor [79]
			9(1H)-Phenanthrenone	41.039	anti-inflammatory [80]

### Direct analysis in real time, time of flight mass spectrometry of shoot and callus extract of *J. procera*

**Fig. 5:**
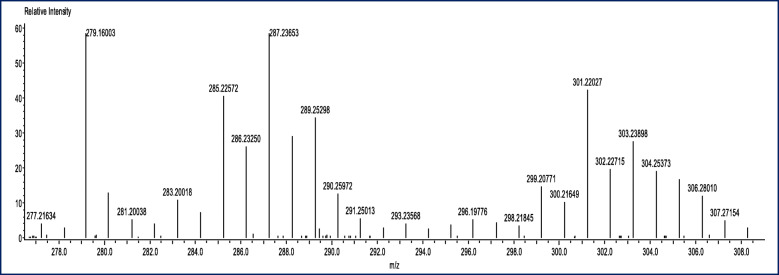
DART TOF-MS of in vitro shoot extract of *Juniperus procera* showing the ferruginol spectrum

**Fig. 6:**
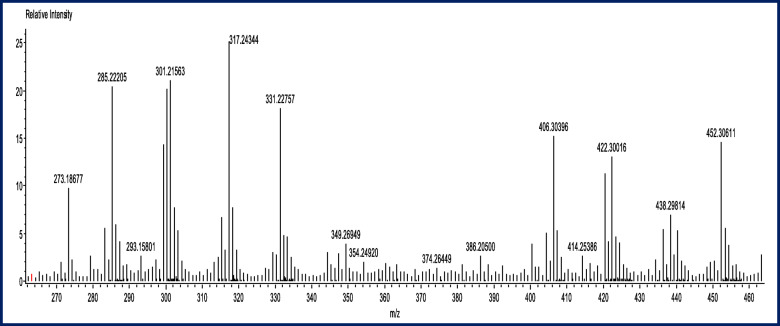
DART TOF-MS of in vitro callus extract of *Juniperus procera* showing the ferruginol spectrum

The in vitro shoot and callus extracts were submitted to characterization by DART-ToF-MS analysis; this has confirmed the result achieved by GC-Ms analysis and thus validated the ferruginol compound showed in Figs. 5-6 and Tables 5 and 6. DART-MS with the characters of rapid quantification, active compounds screening, and has offered a new research tool for herbal medicines to complete the experimental process in a very simple way [85].

**Table 5:** Main constituents (1.25–1.60 min) of in vitro shoot extract of *Juniperus procera* detected by DART-ToF-MS.

N^o^	Experimental mass	Calculated mass	Mass difference (mmu)	Formula	Unsaturation degree	Possible Compound
1	285.22572	285.22184	*3.88*	C_20_H_29_O	6.5	Ferruginol
2	286.23250	286.22966	*2.84*	C_20_H_30_O	6.0	
3	287.23653	287.23749	*−0.96*	C_20_H_31_O	5.5	
4	288.24612	288.24531	*0.81*	C_20_H_32_O	5.0	Podocarp-7-en-3β-ol
5	289.25298	289.25314	*−0.16*	C_20_H_33_O	4.5	
6	290.25972	290.25649	*3.23*	C_19_^13^CH_33_O	4.5	kauren-19-ol

**Table 6:** Main constituents (1.25–1.60 min) of in vitro callus extract of *Juniperus procera* detected by DART-ToF-MS.

N^o^	Experimental mass	Calculated mass	Mass difference (mmu)	Formula	Unsaturation degree	Probable Compound
1	285.22205	285.22184	*0.21*	C_20_H_29_O	6.5	Ferruginol
2	286.22634	286.22519	*1.15*	C_19_^13^CH_29_O	6.5	
3	287.22853	287.22855	*−0.02*	C_18_^13^C_2_H_29_O	6.5	
4	288.22940	288.23190	*−2.50*	C_17_^13^C_3_H_29_O	6.5	
5	414.25386			C_22_H_22_O_8_		Podophyllotoxin

### Identification of Podophyllotoxin

**Fig. 7:**
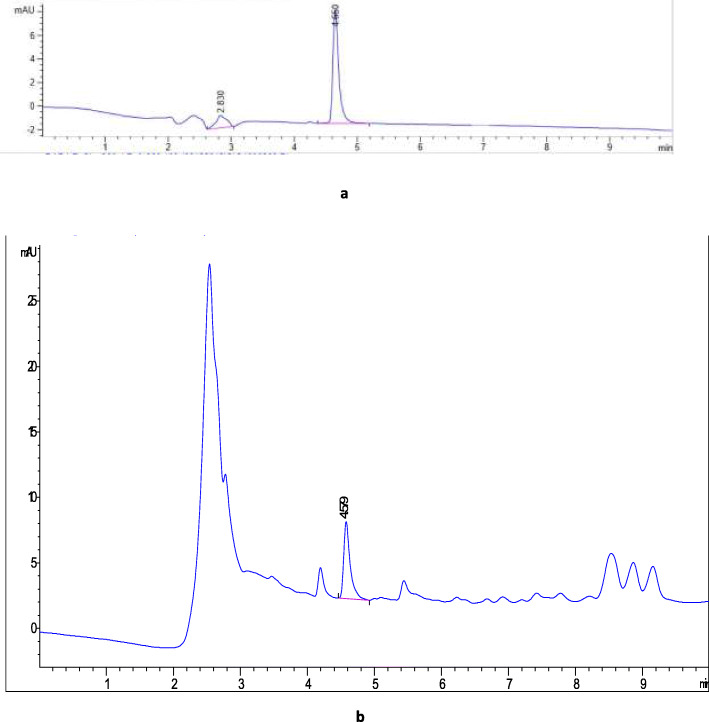
(**a**) HPLC chromatograms podophyllotoxin standard (at 290 nm) (**b**) HPLC chromatograms podophyllotoxin in callus extract (at 290 nm)

**Fig. 8:**
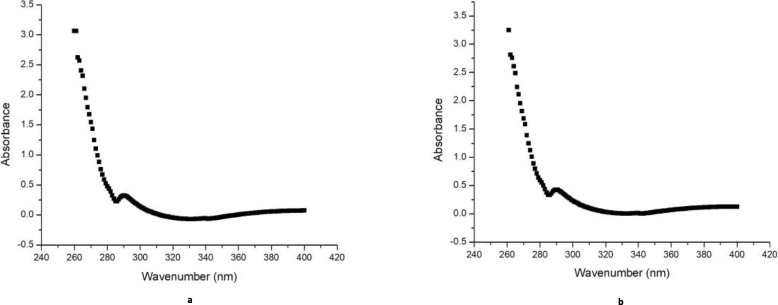
(**a**) UV spectra of Podophyllotoxin standard and (**b**) UV spectra of Podophyllotoxin in callus extract

Podophyllotoxin is secondary metabolites with potent pharmaceutical applications in cancer therapy [86, 87]. However, the availability of podophyllotoxin from its current natural source*, Podophyllum hexandrum Royle* is limited. Hence, alternative sources are urgently needed [86, 88]. In this present study, Podophyllotoxin were initially investigated in callus and shoots of *Juniperus procera* using HPLC and podophyllotoxin standard by comparison of their retention times (Fig. 7, a and b). The result revealed that callus material of *Juniperus procera* is contained novel anticancer agent (PTOX) while not detected in Shoots of *Juniperus procera* in vitro. The callus induction from vegetative part can increase the bioactive compounds or even generate new ones. This is in agreement with findings of [89] who has been stated that, callus cultures *of Byrsonima verbascifolia* (L.) DC. is a new of source of bioactive compounds. The retention time of podophyllotoxin standard was 4.6 min as shown in (Fig. 7, a). So far, the identification of PTOX in callus was confirmed by comparing UV spectra of standard and callus extract. The UV-Visible analysis was performed by SHIMADZU SPECTROPHOTOMETER (UV − 1800) in the range of 200-400 nm. The UV-Visible analysis of PTOX in callus showed that an absorption peak of PTOX at 290 nm (Fig. 8, b) and corresponding well with sigma PTOX standard (Fig. 8, a). Our findings match well with result reported by [90] who has been mentioned that, PTOX UV spectra peak at 290 nm. According to literature, podophyllotoxin has been found in some species of *Juniperus* at low levels in most cases reported by [86]. Furthermore, the result of DART TOF-MS showed that, callus of *Juniperus procera* contained podophyllotoxin (wt. 414.25) Fig. 6.

## Conclusions

To date, there has been no report related to *J. procera* in vitro propagation. Therefore, this study demonstrated the first callus induction and shoot multiplication *of J. procera* indicates that the in vitro propagation of this important, endangered, medicinal plant is possible. We successfully conducted in vitro shoot multiplication and callus induction from *J. procera* seedlings using WPM and a different combination of PGRs. The combination of IAA and BAP at 0.5 μM of both was the best PGR combination on WPM for shoot multiplication and shoot survival rates, while callus induction, quality, and optimization was 0.5 μM of both 2,4-D and BAP. In vitro propagation of *J. procera* creates significant advantages to overcome the difficulties of seeds dormancy for the reproduction of plants, conservation of trees, and getting mass propagation material for pharmaceutical studies. GC-MS and HPLC analysis of shoot and callus extract of *J. procera* has been provided many antitumor and antibacterial constituents, and among them are PTOX and ferruginol. The findings have been confirmed by UV spectrophotometer and Direct Analysis in Real Time, Time of Flight Mass Spectrometry. This result may contribute to the production of in vitro raised plants in large quantities for production of phytochemicals and medicinal constituents (new natural source for an important secondary metabolites), serving also conservation purposes in Saudi Arabia and elsewhere. More research should be conducted on root regeneration of this plant because it will increase the survival rate and conservation of these plants and counteract the difficulties of reproduction from seeds as a consequence of the low germination rate due to seed dormancy, the underdevelopment of seeds, and the problem of dieback. Therefore, in vitro propagation and vegetative reproduction could provide promising solutions.

## Data Availability

The data used or analyzed during the present study are available from the corresponding author/KSU.

## Abbreviations

2,4-D: 2,4-dichlorophenoxyacetic acid
BAP: benzylaminopurine
MS: Murashige and Skoog medium [36]
WPM: Woody plant medium, Lloyd and McCown [35]
B5: Gamborg’s B-5 Basal Medium, devolved by Gamborg, *et. al*. [37]
N6: Chu (N6) Basal Salt Mixture [38]
IAA: Indole Acetic acid
IBA: Indole-3-butyric acid
PGRs: Plant Growth Regulators
PTOX: Podophyllotoxin
GC-MS: Gas chromatography-mass spectrometry
RT: Retention time
DART-ToF-MS: Direct Analysis in Real Time, Time of Flight Mass Spectrometry
